# Successful Approaches for a Red Seaweed Biorefinery

**DOI:** 10.3390/md17110620

**Published:** 2019-10-30

**Authors:** Milena Álvarez-Viñas, Noelia Flórez-Fernández, M. Dolores Torres, Herminia Domínguez

**Affiliations:** Department of Chemical Engineering, Faculty of Sciences, University of Vigo, Campus Ourense, As Lagoas, 32004 Ourense, Spainnoelia.florez@uvigo.es (N.F.-F.); matorres@uvigo.es (M.D.T.)

**Keywords:** red seaweed, cascade processing, biorefinery, phycocolloids, bioactives, energy

## Abstract

Macroalgae have been commercially exploited as food and for the production of phycocolloids, but they also contain compounds with potential in pharmaceutical, nutraceutical, cosmetic, chemical and energetic applications. The biorefinery concept applied to seaweed facilitates the extraction of different constituents ensuring full utilization of resources and generating few residues through a succession of steps. Seaweed biorefineries are less advanced than those based on terrestrial biomass and the design of efficient processes requires further study. This review presents practical successful examples to obtain two or more commercially valuable components from red seaweeds. The selected processes consist on cascading stages of both conventional and alternative techniques to illustrate different possible valorization strategies.

## 1. Introduction

The presence of some unique bioactive components not found in terrestrial biomass makes seaweed an excellent sustainable resource [1]. Seaweeds have been traditionally used as foods and as a source of gelling and thickening agents. Most of the current utilization schemes are based on processing a single seaweed into a single product, whereas the rest of the material is discarded [2]. The valorization of all biomass and co-production of other compounds for the food, feed, pharmaceutical, cosmetic or medicinal industries is desirable [3,4,5,6,7,8]. For an integral utilization of the resources, the sequential separation of all constituents is usually required following a multistage process [1]. This biorefinery concept offers a scheme for the production of high value added components, warrants the maximum utilization of the biomass, reduces the cost of production, offers environmental protection and mitigates climate change impact [3,5,9,10,11].

Interests in macroalgae biorefinery is increasing to solve environmental and economic drawbacks of terrestrial biomass biorefinery, but the biorefineries from seaweed are more recent than from terrestrial biomass and are not mature. Due to the different composition of terrestrial and seaweed biomass, the technologies proposed for one could be not directly applied to another and new developments need to be explored for the utilization of macroalgae [1,7,9,12,13].

Reviews on the potential of macroalgae as biorefinery feedstocks are available, and have been focused on the current trends, different processing options and conversion pathways, products, opportunities, economic considerations, the challenges on the large scale application and the role of seaweed biorefineries in sustainable bioeconomy [3,7,9,10,11,12,14,15]. The present review complements a previous work on the potential of red seaweed and the use of emerging extraction technologies [8], suggesting the interest of an integral valorization of these resources following a biorefinery approach, which is now discussed and presented in more detail and illustrated with practical examples. The objective is to compile information from selected recent successful cascade processing using physical, chemical and biotechnological operations. 

## 2. Red Seaweed

Several red seaweed species are economically relevant because they represent 61% of the total global seaweed production [16]. They are mainly destined to extract agar and carrageenan, but also contain other compounds, in amounts varying greatly with species and seasons. Agar, formed by alternating β-d-galactose and α-L-galactose, can account for up to 57% d.w., and has application in food and pharmaceutical industries, whereas agar oligosaccharides present interesting therapeutic properties [8,11,17]. Agar has higher commercial value than carrageenan, which is a linear polysaccharide made up of a sequence of α-d-galactopyranose and β-d-galactopyranose. Carrageenan accounts for up to 75% seaweed d.w., and can be used for its thickening and stabilizing properties and for its biological activities [11,12,16].

Red seaweed is an excellent raw material for biorefineries, based on their valuable compounds, their content being dependent on species and environmental conditions [4,8,18]. Average ranges are summarized in Table 1. Among macroalgae, red seaweeds contain the highest protein content, up to 45% d.w., and protein digestibility than other seaweeds. Red seaweeds may be used in intact dried form as protein sources in food and feed and the bioactive peptides have diverse actions, such as immunomodulatory, antibacterial, antithrombotic, and antihypertensive effects [19,20]. Seaweed biomass produced with aquaculture effluents contain 2–4-fold more protein than that that grown in wild cultures, because seaweeds assimilate the fish effluents, which are rich in dissolved ammonia and phosphate [21].

Red seaweeds have developed defense mechanisms including the synthesis of photo-protective molecules against UV radiation, such as pigmented compounds (i.e., chlorophyll-a, phycobiliproteins and carotenoids) and mycosporine-like amino acids [22]. They have potential applications as colorants, UV protectors and antioxidants. The chlorophyll a content of red macroalgae, higher than for chlorophyll b, has been reported in the range 0.35-9.8 mg/g d.w. [3,23,24]. These greenish pigments have antioxidant bioactivity and can be converted into compounds with cancer preventive action [25]. The carotenoids of red macroalgae account for 0.2–1.8 mg/g d.w. [3,26], the major being β-carotene, zeaxanthin, lutein and antheraxanthin. Red seaweeds also synthesize phenolic compounds that exhibit free-radical scavenging properties [3,27], accounting for up to 6 mg gallic acid eq/g d.w. [28,29]. Red seaweeds also contain vitamins, with 2.1–2.7 mg pro-vitamin A/g, 0.05–1.54 mg vitamin B2/g, 3.8–4.8 mg vitamin B6/g, 0.4–1.0 mg vitamin B9/g, 2.5–501 mg vitamin C/ 100 d.w., 1.34 mg vitamin E/g d.w. and 4.61 μg α-tocopherol/g d.w. [24,26,29,30]. Phycobiliproteins, present in red macroalgae in the range of <1–125 mg/g d.w., have a great economic potential and can be used in biomedical, food, cosmetics and pharmaceutical applications. Contents in the range 0.8–7 mg R-phycoerythrin/g d.w., 0.02–10 mg phycocyanin/g d.w. have been reported [4,23,31,32].

Despite the low lipid content, these are rich in nutritionally important PUFAs (65.6% of total fatty acids) [33]. Their ω-3:ω-6 ratio is higher than in terrestrial sources, and could be used as nutraceuticals for their anti-hypercholesterolemic, antioxidant, anticancer, antidiabetic, antihypertensive and anti-inflammatory activities [26,33].

Seaweeds present a high content (8–55 g/100 g d.w.) of essential minerals and trace elements [34,35], with high proportions of sulfate (1.3–5.9%). They could be potential ingredients for functional foods, providing a supplementation in deficitary elements in diets [36]. Rhodophycean species contain important macrominerals, 6.1–21.9 g K/100 g d.w., 1.8–8.1 g Na/100 g, 0.2–0.9 g Ca/100 g d.w. and 0.2–0.5 g Mg/100 g d.w. [4,35,37,38], among the trace minerals Fe was the most abundant followed by Zn, Cu and Se [25]. The low Na/K ratio in these seaweeds is beneficial in relation to hypertension [38].

## 3. Seaweed Biorefinery

Marine macroalgae present economic and environmental advantages over terrestrial biomass, including (i) rapid growth rate and photosynthetic efficiency, (ii) no competition for agricultural land, (iii) cultivation in saltwater without needing fertilization, (iv) they contain unique compounds and (v) the depolymerisation of seaweeds before the biological conversion into biofuels is facilitated due to the high carbohydrate and low lignin content [9,12,27]. 

Seaweed biorefinery complies with the sustainable development goals and bioeconomy key principles, mainly food security, sustainable use of resources, reduced climate impact, job generation and competitiveness [15] and with United Nations Sustainable Development Goals related to zero hunger, clean energy and technologies, economic growth, resource-use efficiency, responsible consumption and production and climate action [11]. This approach also allows minimization of residues to a nearly zero waste system, adding higher value to waste fractions, which are reusable and recyclable in a circular economy strategy, increasing the resource efficiency, and giving the highest social, environmental and economic benefits [9,15,51]. Another characteristic of biorefinery is in relation to flexibility and diversity, covering many sectors and producing different outputs using different techniques [9,52]. 

Macroalgae contain phycocolloids, essential amino acids, pigments and polyphenols, useful for the production of high-value products, facilitating the integral utilization of biomass and the coproduction of energy [7]. The production cost of protein, sugar based chemical and inorganics from seaweeds is lower than their market prices, making the biorefinery economically attractive [11]. Since products with highest value should be extracted first and products with lower value after [9], the priority from macroalgae would be the extraction of functional or bioactive components, and then sugars and minerals. When the solid waste fraction can be processed to monomeric sugars, two alternatives are possible, (i) for the production of rare sugars [3], or (ii) utilization of sugars as a carbon source for the bioconversion into biofuels or catalytic conversion into valuable platform chemicals [14]. 

Seaweeds are one potential alternative source for producing energy, ethanol being the most studied final product, due to (i) the possibility of being incorporated into current transportation infrastructure and (ii) to its commercial value as a platform chemical [14,52]. The production of bioethanol from seaweeds residues is an economically attractive option over lignocellulosics because it involves fewer unit operations (hydrolysis and fermentation), demands less energy-intensive chemical pre-treatments, and the protein rich composition of the materials could avoid the use of nutrient supplementation during fermentation. Adapted microorganism strains are required for bioconversion, particularly those with higher galactose metabolism capabilities [52]. The major challenges include the high salt concentrations, causing inhibition of fermentation processes or corrosion and the development of bioprocessing routes towards chemicals with market price greater than bioethanol, such as levulinic acid by controlled acid hydrolysis; 2,5-furandicarboxylic acid by heterogeneous catalytic reaction; succinic acid by tricarboxylic acid cycle or lactic acid by fermentation [1,11]. 

The sustainable use of macroalgal biomass requires management from production, harvesting, processing and packaging to transport and storage. Different sequential steps can be performed: dewatering, extraction, concentration and purification, and final conversion of wastes to energy. Among the extraction options, both conventional and emerging technologies (pressurized solvent extraction, extraction assisted by enzymes, microwaves or ultrasound) could be used. The high water content of seaweeds (ranging from 75 to 90%), is one of the bottlenecks for their sustainable use [53,54]. If possible, the direct utilization of fresh seaweed would offer advantages, since wet biomass is costly to transport and generates polluting effluents. However, since seaweed biomass can deteriorate rapidly, drying is desirable before transportation. Dewatering pre-treatments need to be specific for the type of seaweed to be processed [54,55], and when applied at an early processing stage provides better quality material and decreases both transport costs and associated greenhouse gas emissions [54,55]. Oven-drying, spray-drying, freeze-drying, and microwave-drying are options more energy intensive than sun-drying [56,57,58]. The energy costs of mechanical dewatering are typically an order of magnitude lower than for thermal drying. The pre-treatment before mechanical pressing can be not always appropriate or necessary for all seaweeds and, in some cases, direct pressing is preferable. In energetic applications, such as bioconversion into bioethanol, biohydrogen or methane, direct use of wet feedstocks is more competitive. However, combustion, pyrolysis, and hydrothermal liquefaction and gasification require, in many cases, a dry feedstock [54]. Additionally, the ash content should be considered, whereas pyrolysis can deal with feedstock with high ash content, bioconversion to bioethanol, chemical intermediates or anaerobic digestion are limited by the high ash content [52,59]. Seaweed conversion using cascade conversion should be adapted to local conditions [9]. The processing of fresh biomass should be conducted in the vicinity of origin, and the steady availability of macroalgal wastes and the logistics of biomass transportation to the biogas plant will be important aspects [6]. Process design, with the respective models for mass and energy balances need to be developed, and then environmental life cycle assessment and economic evaluation can provide comparison of multiple processing pathways, technologies, process conditions, and products under equivalent conditions [60]. 

## 4. Biorefinery Strategies

According to their composition, red seaweeds are excellent raw materials for the production of compounds with interest for a variety of applications. Among the potential products from macroalgae in a cascading biorefinery approach, proteins and bioactive compounds are usually preferred for their high value applications; also fatty acids, minerals and phenolics are valuable and demanded fractions. Different strategies have been reported for the utilization of (i) the whole seaweed, either fresh or previously dried, (ii) food wastes and (iii) industrial wastes from the production of phycocolloids. Industrial food wastes, such as the discolored discarded seaweed can be a source for agar production, even with higher yields than from normal seaweeds [61]. A simple sequence could be defined for the energetic valorization of food wastes, such as those from *Gracilaria lemaneiformis* by microwave-assisted acid treatment to yield 16.3% levulinic acid and hydrochar with 45–55% yield and heating values of 19–25 MJ/kg [62]. However, separate valorization of different seaweed components is preferable. Examples of using industrial wastes from protein and phycocolloid extraction and cascade biorefinery are discussed, in increasing order of the number of final products.

### 4.1. Agarophytes

Phycocolloids are one of the major commercial products from seaweed and generate important amounts of waste, because only 15–30% of the total dry mass is used whereas the remaining 70–85% get degraded or drained out as a waste effluent [63]. A number of proposals tried to solve the valorization of the solid residues after agar or carrageenan extraction. These residues could potentially be further processed to generate commercial products, since they are found in a disrupted state, which is advantageous for further microbiological conversion as compared to the initial biomass. When processing the whole seaweed several schemes based on the valorization of the phycocolloid extraction wastes could be proposed, and phycocolloids, with an increasing demand, should be maintained as key products. Alternatively, some red seaweed utilization processes are primarily aimed at the extraction of protein, rich in essential amino acids and can be used as food additives, flavor enhancers and pharmaceutical ingredients. Seaweeds can provide a cheap source of protein, including essential amino acids for undernourished populations and can replace meat in developed countries, being a source of bioactive amino acids, with important roles in food and pharmaceuticals [11].

#### 4.1.1. Agar Extraction Wastes

Agarophytes include the commercially exploited *Gracilaria*, *Gelidium* and *Gelidiella* genus [4]. Depending on the harvest time, the agar yield ranges from 27 to 33% and the manufacturing solid residues, estimated as 15–40% of initial dry biomass, are usually discarded, but still contain important amounts of carbohydrates, in the range 30–70% [5,14,43].

##### Agar-Low Molecular Weight Carbohydrates

**Figure 1:**
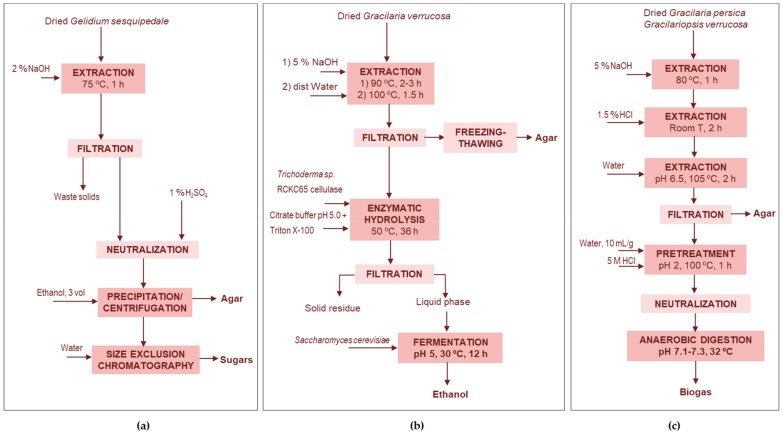
Valorization of the sugar components present in the waste streams after preparation of agar by (**a**) recovering low molecular weight oligomeric sugars [64], (**b**) producing ethanol [43,66] and (**c**) producing methane [44].

Lebbar et al. [64] used the residue after the industrial alkaline extraction of agar from *Gelidium sesquipedale* to obtain low-molecular-weight carbohydrates, in particular glycerol-galactosides, such as floridoside derivatives. Floridoside shows a number of beneficial effects on human health, including antioxidant, anti-inflammatory, neuroprotective and promoter of osteogenic differentiation [64,65], and also presents interest for plant growth [64]. The air-dried *G. sesquipedale* was treated with alkali. After filtration, the alkaline extract was neutralized, vacuum concentrated and agar was separated by precipitation with ethanol (Figure 1a). The ethanolic extract was concentrated by rotary evaporation and subjected to size exclusion chromatography (100–1800 Da) with demineralized water as the eluent; the recovered loe molecular carbohydrate fraction accounted for 8.4% of the algal dry mass. 

##### Agar-Bioethanol

Shukla et al. [43] used the residual pulp from *G. verrucosa* after agar extraction for bioethanol production (Figure 1b). These solids were enzymatically hydrolyzed to yield a maximum sugar concentration of 75.8 g/L with 63% saccharification yield and the bioconversion of this enzymatic hydrolysate using *Saccharomyces cerevisiae* led to 27 g/L ethanol with a yield of 0.49 g/g. Both the enzymatic hydrolysis and fermentation processes were successfully scaled up.

##### Agar-Methane

Hessami et al. [44] reported the utilization of the industrial wastes from the extraction of agar from both *Gracilaria manilaensis* and *Gracilariopsis persica* for methane production (Figure 1c). The methane yields were higher than from the whole seaweeds, reaching 70% and 62% of the theoretical yield, respectively. The dried red seaweed, was pretreated with alkali, the solid phase was washed with tap water and extracted with 1.5% HCl at room temperature. The separated solid phase was washed and the agar was extracted with distilled water. Following agar extraction, the carbohydrate contents were reduced by >80% for both species, the lipid content by more than 90%, and protein by 70–90%. The residual solids were pretreated under mild acid conditions before neutralization and anaerobic digestion. The mild acid pre-treatment did not significantly affect the carbohydrate and the fat content, but the protein content was lowered to 7–14%, due to the loosened structures caused by the mild acid conditions. The dilute acid pretreatment of *G. manilaensis* and *G. persica* residues improved the methane yield by 47% and 60%, compared to the untreated material. This strategy also avoids depositions in anaerobic digesters, caused by the high levels of complex sulphated polysaccharides, halogens and salinity, which are associated to the use of whole seaweed. 

##### Agar-Biochar

Méndez et al. [67] used an industrial macroalgal waste from the agar extraction as a precursor for the production of meso-macroporous hydrochars by hydrothermal carbonization, with a yield of up to 60%, which doubled the value attained with conventional char. The hydrochar obtained after 6 h at 230 °C retained, to a large extent, the vegetal structure of the wastes and presented a similar heating value to that of lignite. Roberts et al. [68] produced iron-based sorbents from the waste biomass that remains after the commercial extraction of agar from farmed *Gracilaria edulis* biomass treated with a ferric solution, then converted to biochar through slow pyrolysis. 

#### 4.1.2. Protein Extraction Wastes

##### Phycobiliproteins-Ethanol

**Figure 2:**
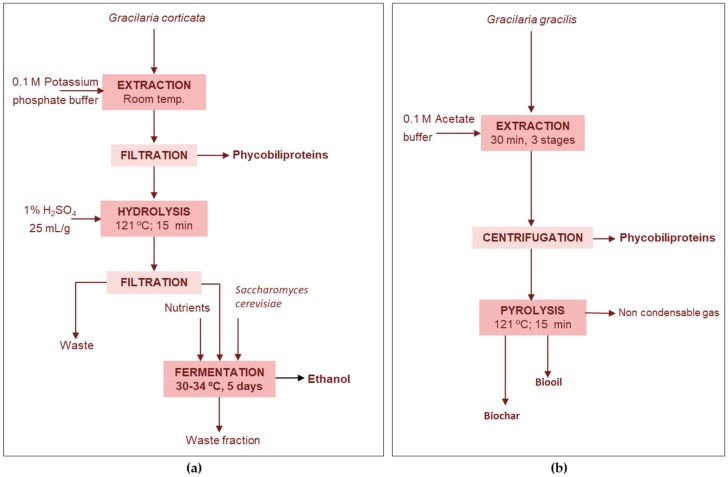
Example of valorization of the solid remaining after phycobiliproteins extraction (**a**) for ethanol production from *G. corticata* [69] and (**b**) for bio-oil and biochar from *G. gracilis* [42].

Sudhakar et al. [69] proposed the use of the spent biomass from *Gracilaria corticata* remaining after pigment extraction with 0.1 M potassium phosphate buffer. This waste fraction contained 19% polysaccharides, 1% protein and 0.2% phenolics, and was destined to the production of ethanol, after an acid pretreatment (Figure 2a). This alternative offered higher reducing sugar yield (18%) than the direct use of the industrial spent biomass after phycocoloid extraction (1.7%). The hydrolysates, separated by filtration, once changed the pH to 5.3 and supplemented with nutrients were used for fermentation with *S. cerevisiae* to produce ethanol with a yield of 0.02 g/g, lower than that obtained from a standard media supplemented with yeast-peptone-dextrose and with d-galactose. 

##### Proteins-Bio-Oil-Biochar

A cascade biorefinery approach for *Gracilaria gracilis* consisted on the extraction of phycobiliproteins (7 mg R-phycoerythrin/g d.w., 3.5 mg allophycocyanin/g d.w. and 2 mg phycocyanin/g d.w.) and further pyrolysis of the residue to produce bio-oil and biochar (Figure 2b). The bio-oil yield was 65 wt% at 500 °C, the high nitrogen content prevents its use as a biofuel and would required a denitrogenation stage. Biochar yields ranged between 26.5% (600 °C) and 33.0% (400 °C) and inorganic elements such as P, K, Ca, Fe and Mg were present in the solid product [42]. 

##### Lipids-Fertilizers-Agar-Bioethanol 

**Figure 3:**
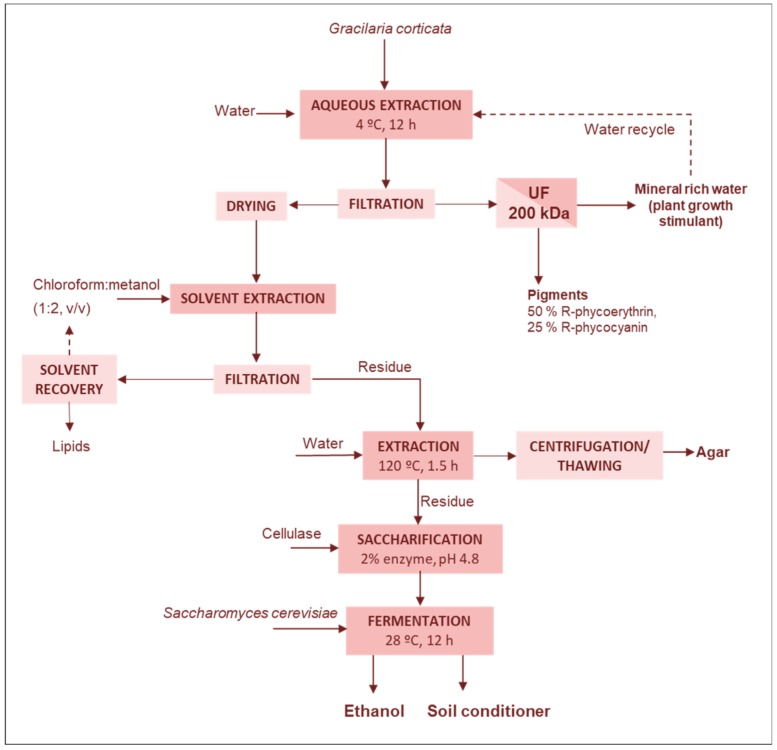
Scheme of the cascade process proposed for the extraction of phycobiliproteins, minerals, lipids, agar and for the production of ethanol from *Gracilaria corticata* fresh biomass [5,53].

One of the most complete biorefinery approach for agarophytes has been proposed to process the fresh biomass of *Gracilaria corticata* [5,53]. The cascading process allows for the complete utilization of seaweeds, recycling solvents leaving no solid waste, and recovering six different products, with an estimated market value of fourfold the processing cost and raw material [53]. The fresh seaweed was homogenized in water and extracted at 4 °C during 12 h to solubilize the pigments, which could be recovered by i) 30% ammonium sulfate precipitation of ii) by ultrafiltration (Figure 3). From one tonne of fresh biomass 0.3–0.7 kg of R-phycoerythrin and 0.1–0.3 kg of R-phycocyanin were obtained. Up to 96% R-phycoerythrin and 92% R-phycocyanin were recovered in the retentate, with a final purity of 50% and 25%, respectively. The permeate stream can be recycled up to three times to the previous extraction stage, with a progressive increase of mineral content with each recycle step. This mineral rich permeate could be used as a plant growth stimulant used in foliar applications.

The residual biomass after pigment extraction was dried and used for extraction of lipids with a chloroform–methanol (1:2 *v*/*v*) mixture, which could be recovered and reused. The crude lipid extraction yield, 1.41% d.w., was comparable to that attained from direct seaweed extraction. The high PUFA fraction (65.6%) of the total fatty acids could recommend its use for food and nutraceutical applications. The dried solid residue after lipid extraction was used for agar extraction with water at 120 °C, providing yields and gelling properties comparable to those attainable when processing the initial seaweed. The residual solid remaining after agar extraction, representing 23% of the initial dry biomass, was hydrolyzed with a commercial cellulase to yield 0.27 g reducing sugars/g residue. Fermentation of hydrolysate with *Saccharomyces cerevisiae* produced ethanol with 92% conversion efficiency and a yield of 12.7% based on residual mass. The residue obtained after fermentation accounted for 12.4% of the raw material, the C:H:N:S ratio was 42.8:6:9.7:0.75 and could be proposed as a soil conditioner. This computed yield data from small-scale biorefinery trials confirmed that a tonne of fresh biomass provides: 0.3–0.7 kg of R-phycoerythrin, 0.1–0.3 kg of R-phycocyanin, 1.2–4.8 kg of lipids, 28.4–94.4 kg of agar, 4.4–41.9 kg of cellulose and 3.1–3.6 kL of mineral solution; and after enzymatic hydrolysis and bioconversion would yield 1.8–17.4 kg of ethanol [53]. 

### 4.2. Carragenophytes

#### 4.2.1. Carrageenan Extraction Wastes

Carrageenan is the seaweed hydrocolloid with the largest sales volume, more than double than alginate and triple than agar [70]. The carrageenan yield can account for 32% of the dry weight of the seaweed, but the amount generated of residual fractions, approx. 30% of the initial material, and the increasing carrageenan production make necessary the valorization of the waste fractions [45,70]. Both the native κ-carrageenan and the leftover fraction successfully reversed obesity and related metabolic syndromes, such as the raised body fat percentage and serum cholesterol level, the increased adipocytes size, the abnormal levels of adipocytokines, and the gut dysbiosis [71]. Despite this direct utilization could be interesting, the generation of high volumes of this waste fraction makes necessary other uses that could consume larger amouts of waste produced.

##### Agricultural Bio-Stimulant and Carrageenan

**Figure 4:**
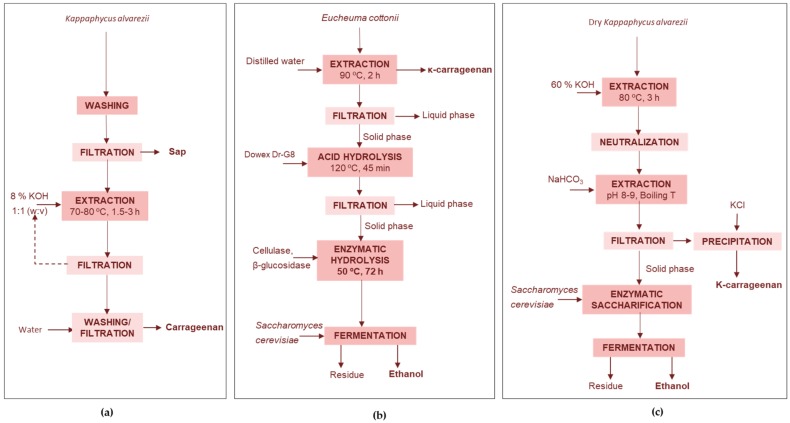
Examples of (**a**) extraction of plant bio-stimulant and semi-refined carrageenan from *K. alvarezii* [72], and production of carrageenan and ethanol from (**b**) *E. cottonii* [74] and (**c**) from *K. alvarezii* [76].

Shanmugam and Seth [72] proposed the extraction of an agricultural bio-stimulant from the fresh biomass of *K. alvarezii* before the production of semi-refined carrageenan in a biorefinery process that avoids a previous drying stage (Figure 4a). They confirmed the viability at a large, pre-commercialization pilot-scale, processing 10 t of fresh biomass. The total recovery of the agricultural bio-stimulant and carrageenan from fresh material processed at different seasons ranged from 1.96 to 2.37% and from 2.43 to 4.16%, respectively. 

For the alkaline treatment before the production of carrageenan, most processes use a KOH treatment stage. Eswaran et al. [73] also claimed a different alternative consisting on using Ca(OH)_2_ at 107 °C and the precipitation of carrageenan with isopropyl alcohol, providing a product with improved gel strength. The integrated process proposed for *Kappaphycus alvarezii* produced both κ-carrageenan (2.5–4.5 t) and a liquid seaweed fertilizer (60–80 t) from 100 t of fresh biomass. The granular residue obtained from the fresh seaweed was a superior raw material than the dried whole seaweed for κ-carrageenan, because it is less bulky, easy to transport, store and handle, has low color and a high κ-carrageenan content.

##### Carrageenan-Ethanol

Tan and Lee [74] proposed the use of the seaweed solid wastes remaining after the extraction of κ-carrageenan from *E. cottonii*. The wastes were processed by enzyme hydrolysis with a cocktail of cellulases and bioconverted with *Saccharomyces cerevisiae*, either in separate stages or by simultaneous saccharification and fermentation process (Figure 4b). Whereas in the first strategy up to 99.8% glucose yield was obtained and the subsequent fermentation yielded 55.9% bioethanol, the simultaneous strategy yielded 90.9% bioethanol in a simple one-step procedure saving time, cost and energy consumption.

Meinita et al. [70] produced ethanol using the solid wastes from the carrageenan extraction of *Kappaphycus alvarezii* (Figure 4c). They used a sequence of hydrolysis stages, the first with sulfuric acid and the second with a mixture of commercial cellulases to yield a solution with 18 g galactose/L and 26 g glucose/L. The formation of 5-hydroxymethylfurfural (HMF) and levulinic acid after enzymatic hydrolysis was low and the hydrolysate was bioconverted to ethanol using *Saccharomyces cerevisiae* ATCC 200062, which yielded 69% of the theoretical ethanol value. The by-products resulting from bioethanol production, with important amounts of organic matter and minerals, can be used as soil fertilizer or for pet food formulation. 

Another alternative consisted on the extraction of carrageenan, with a yield dependent on the operation scale (26–31%, *w*/*w*), then the acid hydrolysis (0.9 N H_2_SO_4_, 100 °C, 1 h) and further saccharification. The hydrolysate was then neutralized with lime and the filtrate was desalted by electrodialysis and converted to ethanol by *S. cerevisiae* with 80% conversion yield, although 50% of the total organic carbon initially present remained unutilized [75]. 

Paz-Cedeno et al. [45] applied the integrated enzymatic and acid hydrolysis of the insoluble by-product from carrageenan extraction to obtain a hydrolysate rich in monomeric sugars, which is not suitable for human consumption, but can be used in feed manufacturing. The galactan fraction of the by-product is poorly hydrolyzed by commercial cellulolytic enzymes and the resulting hydrolysate forms a gel at 30 °C, which causes difficults for a subsequent fermentation. These authors proposed the use of mixtures of commercial enzymes and achieved almost 100% of glucan and 30% galactan conversion, this latter requiring higher enzyme loadings. The mild-acid treatment after enzymatic hydrolysis increased the glucose and galactose concentrations, without raising the inhibitors content and avoiding the formation of a gel structure. The yield of biomass treated with KOH was approximately 72.4%. The major components were galactan, glucan and less than 3% proteins. Nevertheless, after the enzymatic hydrolysis of the by-product, there was still a gel formation in the hydrolysate. 

#### 4.2.2. Processing of the Whole Seaweed

##### Biochar and Sugars

Teh et al. [40] designed a process based on dilute sulfuric acid hydrolysis at 160 °C during 10 min with microwave-assisted heating to produce biochar and sugars from *Eucheuma denticulatum*. Compared to the raw macroalga, biochar qualities were improved with increased carbon content and lower ash and moisture contents. The calorific value of the biochar could be intensified and 39% of energy yield was recovered. Apart from producing biochar, the highest total reducing sugars were 51 g/L, representing 75% yield, with a low by-product concentration (0.2 g HMF/L), since this compound could be further degraded to organic acids, namely levulinic acid and formic acid. The acid treatment at 170 °C increased the cellulose content of the solids from 9.1% in the raw material to 41.0% at 0.2 M, but the reducing sugars were higher when the macroalga was treated with a lower acid concentration. Macroalgae have higher ash content (23%) than lignocellulosic biomass (3%) and microalgae (9%) and the hydrolysis process can improve the fuel properties by reducing the ash content to 13%. Upon hydrolysis, solid residues are very porous, thus aqueous soluble components including reducing sugars tend to precipitate on the pores of the solid and the contents and reactivity of aqueous solubles in thermal process limit the yield of biochars. The aqueous soluble components of biochars are easily metabolized by soil microbes, providing an additional fertilizer effect. 

##### Bioactives-Carrageenan

**Figure 5:**
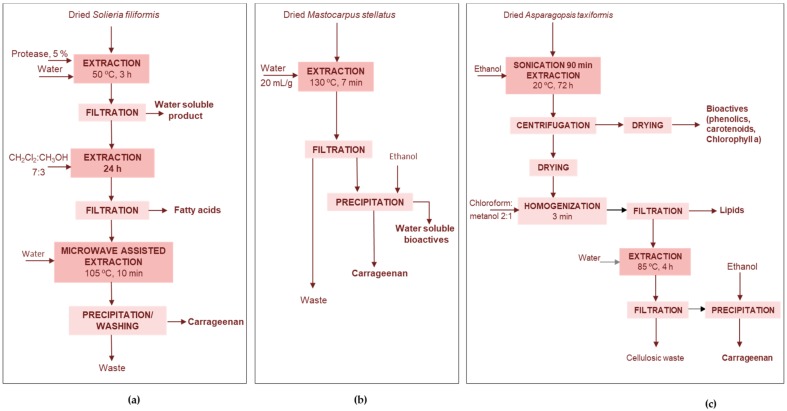
Processes for the extraction of (**a**) three valuable fracctions from *Solieria filiformis* [16], (**b**) carrageenan and water soluble bioactives from *Mastocarpus stellatus* [77], (**c**) carotenoids, lipids, carrageenan and cellulose from *Asparagopsis taxiformis* [25].

Peñuela et al. [16] proposed the utilization of *Solieria filiformis* biomass from integrated multi-trophic aquaculture (Figure 5a). Using an enzymatic-assisted extraction and microwave-assisted extraction process, three products were successfully recovered: (i) a water-soluble extract rich in proteins and sulfated polysaccharides suitable as food supplement, (ii) a lipid fraction with 13.4% polyunsaturated fatty acids with ω6/ω3 ratio 0.9 and (iii) a pure carrageenan with antiviral action against *Herpes simplex* comparable to the commercial antiviral acyclovir. 

Torres et al. [77] proposed the non isothermal autohydrolysis at a final heating temperature of 130 °C for the extraction of hybrid carrageenan with higher extraction yields and improved rheological properties, in terms of strength of viscous and elastic moduli of the derived gels, when compared with those obtained from commerical kappa and iota mixtures at similar ionic strength and equivalent biopolymer content. In addition to the carrageenan fraction, a soluble extract was obtained, containing 16–33% protein at 100–150 °C, and 1–5% phenolic compounds at 90–130 °C and with antiradical properties corresponding to 10 mg Trolox eq/g extract at 130 °C. The antiradical properties were three times higher when the processing temperature was 70 °C and 190 °C.

Biorefining can be useful to enhance the nutritional and health-promoting properties of seaweeds for feed applications [27]. Schiener et al. [78] reported that biorefining resulted in the removal of inorganic elements such as sodium, potassium and chloride. The total solid and ash content in *Palmaria palmata* was reduced, although some divalent metals (Fe, Zn, Si) were accumulated and iodine content was increased. The arsenic removal and the protein concentration in the residue could be increased by more than two-fold, therefore, increasing the nutritional value by an increase in total amino acids and fatty acids. 

##### Iodine-Lipids-Carrageenan-Cellulose

Nunes et al. [25] proposed a sequence of extraction stages to obtain different components from *Asparagopsis taxiformis* (Figure 5c). Despite the extraction with water provided higher yields (32%) than ethanol (19%), this latter was selected, because the extracts presented the highest iodine (3.4%), total phenolic (1.7%) and chlorophyll a (45.9 mg/100 g d.w.) contents. This iodine rich bioactive extract could be used as a nutraceutical supplement, with reducing (2 g ascorbic acid equiv./100 g d.w.), chelating (IC_50_ = 5.3 mg/mL), and antiradical (IC_50, DPPH_ =1.5 mg/mL) properties. The remaining residue was used to obtain lipids (2.0 g/100 g d.w.), carrageenans (21.2 g/100 g d.w.) and cellulose (23.8 g/100 g d.w.). The highest total flavonoid content could be obtained with ethyl acetate (24.2 g quercetin eq./100 g), and total carotenoids content highest value was obtained with methanol (36.23 mg/100 g d.w.). 

##### Carrageenan, Ethanol, Biofertilizer, and Biogas

**Figure 6:**
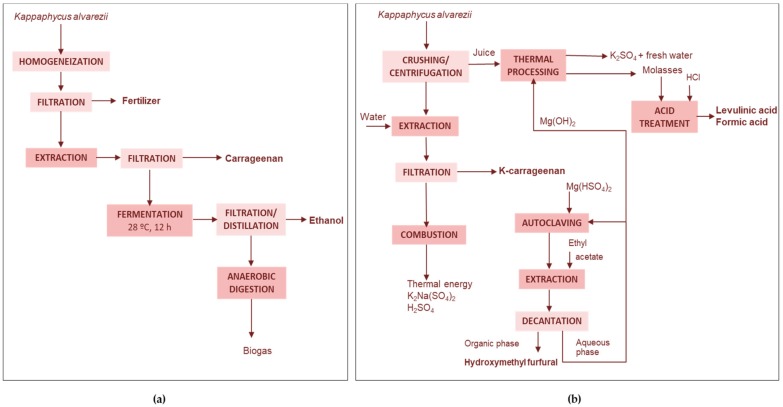
Flow diagram of the biorefineries for (**a**) the co-production of fertilizers, carrageenan, ethanol, and biogas. From fresh *K. alvarezii* [73,79] and (**b**) different chemicals from fresh *Kappaphycus alvarezii* [38].

Eswaran et al. [73] claimed different biorefinery schemes for *Kappaphycus alvarezii*. Ingle et al. [79] reported the production of bioethanol, carrageenan, fertilizer and biogas from this seaweed. They initially obtained 67% sap yield and the remaining residue was subsequently used for carrageenan extraction (12 g/kg dry algae), and ethanol production (77.6 g/kg dry algae), similar values to those from non-extracted algae (Figure 6a). In order to improve the bioethanol yields, these authors proposed a two-step fermentation approach, using *S. cerevisiae* in a first step to convert seaweed biomass and *Escherichia coli* in a second step to bioconvert the fermentation leftovers and the *S. cerevisiae* biomass from the first step. The waste stream from ethanol production was used as a feedstock for bioenergy production by anaerobic digestion followed by the conversion of the biogas to energy for the process using a combined heat and power system. 

##### Fertilizers and Chemicals

Another complete biorefinery process for the production of different compounds from *Kappaphycus alvarezii* has been developed by Mondal et al. [38] (Figure 6b). The initial crushing and filtration separated a 70% (v/w) sap rich in plant growth hormones (indole acetic acid, kinetin, zeatin and gibberellic acid), with a high content of potassium (21 g/L), bromide (41 mg/L), iodide (1.54 mg/L) and iodate (0.44 mg/L). The remaining granular biomass (4% of the fresh material) was used for κ-carrageenan extraction with seawater. Carrageenan was used as for the production of HMF using Mg(HSO_4_)_2_ as catalyst in a reaction also generating galactose which remained as a co-product in the aqueous phase. The aqueous phase was recycled up to 10 times by maintaining a constant acid strength, and was used for the recovery of K_2_SO_4_. Bipolar electrodialysis of seaweed juice was carried out for generation of HCl and KOH. The galactose co-produced during HMF synthesis was used to produce levulinic acid. The stream was neutralized, desulphated with CaCl_2_, acidified with concentrated HCl and reacted in an autoclave under similar conditions to those for HMF synthesis. 

From 1 t of granular biomass the following products could be thus obtained: 0.18 t HMF, 0.056 t levulinic acid, 0.020 t formic acid, 0.27 t K_2_SO_4_, 68.93 m^3^ leaf spray and 5.77 m^3^ pure water. In addition, the energy required for the process could be obtained from the controlled combustion of granules, which also yielded H_2_SO_4_ and a second fertilizer [80].

### 4.3. Porphyran Rich Seaweeds

#### Phycocolloids-Peptides

**Figure 7:**
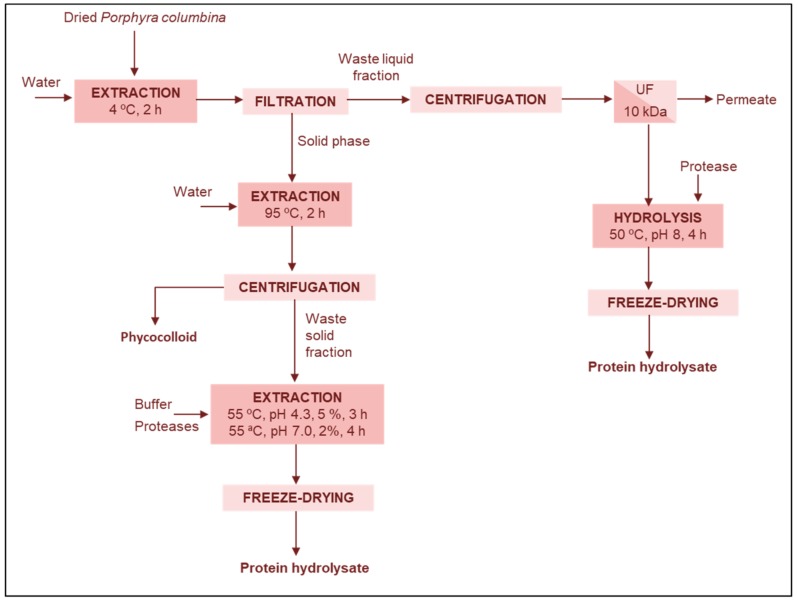
Flow diagram of the aqueous extraction of a protein and a phycocolloid fraction from *Porphyra columbina* [20,48,49].

During the traditional method to obtain phycocolloids from seaweeds using a sequence of extraction steps with cold and hot water, the cold water extract has no phycocolloids but is rich in proteins and is considered a waste, as well as the residual cake after phycocolloid extraction. Cian et al. [20,48,49] proposed different schemes for valorization of these waste streams, including (i) recovery of the proteins from the liquid waste for the formulation of films, (ii) recovery and hydrolysis of the proteins present in the liquid waste and (iii) hydrolysis of the solid waste (Figure 7) 

Cian et al. [49] proposed the utilization of the phycobiliproteins discarded in the cold water stream before the phycocolloids extraction from *Porphyra columbina*, because its excellent antioxidant properties could be of interest for the preparation of edible films formulated with different ratios of proteins and phycocolloids. The phycocolloid fraction extracted and used without further purification steps offers a low cost potential source for producing an edible film, with excellent mechanical properties. The phycobiliprotein fraction exerted a plasticizing effect on the phycocolloid matrix, and conferred antioxidant capacity. The films prepared by mixing different proportions of these two fractions showed intermediate properties, which correlated with its formulation.

Cian et al. [20] proposed the enzymatic hydrolysis of the proteins in the cold water removed from *P. columbina*. The degree of hydrolysis caused by proteolytic enzymes, trypsin, alcalase or a sequential combination of both, was in the range 43.5–64.3%. The hydrolysates were enriched in low molecular weight peptides with a high content of aspartic, glutamic, alanine and a relatively high content of threonine, leucine and proline. They showed immunosuppressive effects on rat splenocytes, also antihypertensive activity (>35% of ACE inhibition) and antioxidant capacity (DPPH, TEAC, ORAC and copper-chelation) more potent than before hydrolysis. The cold water extract presented a low content of epicatechin, p-coumaric acid, quercetin and kaempferol, but after acid hydrolysis, which breaks protein-polyphenol interactions and allows depolymerization of phenolic compounds, the content of gallic acid, catechin and epicatechin increased [20].

Cian et al. [48] proposed the valorization of the residual cake remaining after the extraction of phycocolloids from *Porphyra columbina*. The solid waste, containing 27% protein and 10.7 mg gallic acid eq/100 g, has low economical value as animal feed due to its low protein digestibility (59%). In a scalable process consisting on an enzymatic hydrolysis (fungal protease concentrate and commercial protease) to obtain bioactive peptides and polyphenols, the degree of hydrolysis was 15%. Whereas the residual cake presented components with molecular weight around 20 kDa, the hydrolysate exhibited two main peaks (around 5.5 kDa and < 500 Da), with Asp, Glu, Ala, and Leu. The hydrolysate showed higher protein solubility than the residual cake, as well as ACE inhibition (45.6%), copper-chelating activity (97.5%) and radical scavenging activity (IC_50, DPPH_ = 0.9 g/L and IC_50, ABTS_ = 1.0 g/L), with a Trolox equivalent antioxidant capacity (TEAC) value of 2.8 mmol Trolox/L. These properties were attributed mainly to low molecular weight peptides and polyphenolic compounds released during proteolysis, which altered the interaction between peptides and polyphenols.

## 5. Conclusions

Seaweeds are receiving increasing global attention as potentially sustainable ingredients in food, feed, cosmetics, pharmaceutical applications, or as raw materials for chemical, biomaterials and energetic products. The integral valorization of the components following a biorefinery approach would represent the most sustainable processing alternative. A number of multistage multiproduct designs have been reported and compiled with the aim of providing an updated summary of possibilities for the rational utilization of this resource. Additionally, the waste fractions after phycocolloid extraction can be a valuable raw material for the production of proteins, oils, minerals, chemicals and biofuels. It is expected that this review encourages both researchers and industrial users to apply this type of cascade biorefinery schemes and to improve them in economically viable processes in future. 

## Figures and Tables

**Table 1:** Average composition or ranges of widely used red seaweeds, expressed in% d.w. (5.3–12.1% moisture) and biological properties of the major components.

Seaweed Genus	Carbohydrates	Protein	Lipids	Minerals	Phenolics	Chla/Car (mg/g)	Ref.
*Asparagopsis*	40.5	17.6	6.62	23.76	0.06	0.03/0.01	[39]
*Chondrus*	50.20–64.6	6.7–18.1	2.46–4.20	19.8–22.8	0.04–0.43	0.06/0.02	[28,29,39]
*Eucheuma*	65.82	4.9	0.10	17.3			[40]
*Galaxaura*	10.28	2.80	1.43	84.36			[39]
*Grateulopia*	73.37	4.9	2.97	16.60	0.02	0.18/0.13	[39]
*Gelidium*	23.5–25.2 ^2^; 10.6–12.2 ^3^	18.4–19.3	1.1–1.3				[4]
*Gelidiella*	24.5^2^; 9.8^3^	14.9	1.4				[4]
*Gelidiopsis*	11.4^3^	17.6	1.3				[4]
*Gigartina*	29.31 ^1^	15.6	0.57	34.56			[41]
*Gracilaria*	24.8–78.7; 11.2–56.6 ^2^; 3.8–6.1 ^3^	0.6–45.0	0.3–7.1	7.4–40.3		0.001–0.06/0.001–0.007	[4,24,26,30,31,42,43,44]
*Graciliaropsis*	77.7	10.5	0.8				[44]
*Kappaphycus*	57.2	2.6	5.2	15.8			[45]
*Mastocarpus*	60.4; 31.70 ^1^	12.1–21.4	0.39	15.6–24.9			[41,46]
*Nemalion*	31.43	3.80	2.17	60.64	0.06	0.09/0.01	[39]
*Solieria*	18.8–22.5	8.1–8.3	2.1–2.5				[16]
*Palmaria*	31.7–59.0	10.7–31.4	4.9–12.9	9.0–23.7	0.48–0.55		[28,29]
*Porphyra*	39.0–64.0^1^	22.3–53.9	1.3	0.5–16.4	0.27–0.35	0.18–0.35/0.03–0.06	[20,23,29]
Properties	AC, AT, AV, Ave, FP	AO, AH	AAt, ACh, AD, AH, AI, AP, AT, IS	DMS	AAt, AI, AO, AP, AT	AI, AO, AT	
References	[8,11,17,47]	[19,20,48,49]	[26,33,35,50]	[36]	[3,27]	[25]	

^1^ TDF: total dietary fiber; ^2^ Agar; ^3^ Cellulose. Chl a: Chlorophyll a; Car: carotenoids; DMS: dietary mineral supplement; FP: functional properties; AC: anticoagulant; AAt: anti-atherosclerotic; ACh: antichlolesterolemic; AD: antidiabetic; AH: antihypertensive; AI: anti-inflammatory; AO: antioxidant; AP: Antiproliferative; AT: antitumoral; Ave: Antivenom; AV: antiviral; IS: immune stimulant.
